# INTEROCEPTIVE ACCURACY ENHANCES DECEPTION DETECTION WITH GREATER AGE

**DOI:** 10.1093/geroni/igad104.3714

**Published:** 2023-12-21

**Authors:** Natalie Ebner, Amber Heemskerk, Tian Lin

**Affiliations:** University of Florida, Gainesville, Florida, United States; University of Florida, Gainesville, Florida, United States; University of Florida, Gainesville, Florida, United States

## Abstract

Financial exploitation of older adults is a growing concern, with millions of seniors scammed annually, costing billions, and resulting in devastating consequences for health, independence, and well-being. Exploitation risk is exacerbated in Alzheimer’s disease and related dementias (AD/ADRD). Detecting deception is challenging, especially for older adults who tend to believe others are truthful. Understanding the neurobiological mechanisms underlying this susceptibility is essential to design effective intervention toward reducing exploitation in aging. In this research we demonstrate the importance of interoceptive awareness─the ability to interpret body signals─in deception detection in older adults. Seventy-six young (18-34 years) and 74 older (53-82 years) adults completed a heartbeat counting task to assess their interoceptive awareness. They also engaged in deception detection paradigms across two distinct, ecologically valid tasks: i) a lie detection task in which they made veracity judgments of genuine and deceptive individuals, and ii) a phishing email detection task to capture accuracy in online deception detection. Greater interoceptive awareness was associated with greater lie detection accuracy. Furthermore, with greater chronological age among older adults, greater interoceptive awareness was associated with better accuracy in both detecting deceptive individuals and phishing emails. These findings support interoceptive awareness as a relevant factor for interventions aimed at enhancing deception detection abilities in aging. Project results will advance insight into the neurobiology of deception detection to inform effective translation into intervention in mitigating exploitation risk in aging and eventually ADRD.

